# Supplementary material for: The first national survey of antimicrobial use among dentists in Japan from 2015 to 2017 based on the national database of health insurance claims and specific health checkups of Japan
Source: PLoS One. 2020 Dec 28;15(12):e0244521. doi: 10.1371/journal.pone.0244521 (PMC7769482; doi:10.1371/journal.pone.0244521)
Supplement: Attachment — Submitted filename: PONE-D-20-31740_Response to Reviewers.docx [file pone.0244521.s001.docx]

[November 26, 2020]

Simon Clegg, PhD

Academic Editor

*PLOS ONE*

Original submission date: Oct 9, 2020

Re: PONE-D-20-31740

Dear Dr. Clegg,

Thank you very much for inviting us to submit a revised draft of our manuscript entitled “**The First National Survey of Antimicrobial Use Among Dentists in Japan from 2015 to 2017 Based on the National Database of Health Insurance Claims and Specific Health Checkups of Japan**”. We really appreciate the time and effort you and each of the reviewers have dedicated to providing insightful feedback.

To facilitate your review of our revisions, we provide below our point-by-point responses to the questions and comments delivered in your letter dated Nov 11, 2020.

We hope that the revised manuscript is now acceptable for publication in *PLOS ONE*, and will be glad to address any further questions that you may have.

Sincerely,

Akane Ono and Masahiro Ishikane (Co-first authors)

Yoshiki Kusama (Corresponding author)

AMR Clinical Reference Center, National Center for Global Health and Medicine Hospital

Postal address: 1-21-1 Toyama, Shinjuku-ku, Tokyo, 1628655, Japan

Tel: +81-3-6228-0062

Fax: +81-3-6228-0420

Email address: [stone.bagle@gmail.com](mailto:stone.bagle@gmail.com)

**Editor's Comment:**

*Thank you for stating the following in the Competing Interests section:*

*"Yuichi Muraki received an honorarium for lecturing from Pfizer Japan, Inc. The other authors have no conflicts of interest to declare."*

*Please confirm that this does not alter your adherence to all PLOS ONE policies on sharing data and materials, by including the following statement: "This does not alter our adherence to PLOS ONE policies on sharing data and materials.” (as detailed online in our guide for authors http://journals.plos.org/plosone/s/competing-interests). If there are restrictions on sharing of data and/or materials, please state these. Please note that we cannot proceed with consideration of your article until this information has been declared.*

Thank you for pointing this out. We confirm that the statement does not conflict with our adherence to *PLOS ONE* policies, and have added "This does not alter our adherence to *PLOS ONE* policies on sharing data and materials.” to the cover letter as instructed.

**Reviewer #1’s Comments:**

*1. If possible, the reasons for the Japanese preference for cephalosporins should be discussed and there should be more discussion and comparison with other countries regarding the most widely used beta lactic alternatives.*

Thank you for your thoughtful and constructive feedback regarding our manuscript.

We are grateful for this important suggestion. A possible reason for the preference for cephalosporins in Japan’s medical facilities may be the active promotion of these antimicrobials by pharmaceutical companies. A recent study reported that the top-selling antimicrobials in Nepal were those with a higher number of promotional activities [21]. Although such activities have since diminished in Japan, the situation was similar to that of Nepal until only 10 years ago. These promotions may have contributed to the high adoption rate of cephalosporins in medical facilities in Japan when compared to those in other countries. In fact, we have previously shown in an analysis of 31 Japanese hospitals that all the study hospitals had adopted the use of oral third-generation cephalosporins [22]. Furthermore, most of these cephalosporins were Japanese-origin drugs that are not commonly used outside of Japan. Based on the results of our previous questionnaire-based study on AMU among dentists in Japan [31], we are currently conducting a nationwide questionnaire-based epidemiological study to determine the factors that influence prescriptions of third-generation cephalosporins among 1700 dentists throughout the country. We have added this information to the Discussion section.

(Before: Discussion, Page 14, Line 201)

These results corroborate those of a previous cross-sectional questionnaire-based study on dentists at community clinics in Japan [20].

(After: Discussion)

These results corroborate those of a previous cross-sectional questionnaire-based study on dentists at community clinics in Japan [20]. The frequent use of cephalosporins in Japan may be due in part to the active promotion of these antimicrobials by pharmaceutical companies. A study conducted in Nepal showed that the top-selling antimicrobials were those that were subjected to intensive promotional activities [21]. Although such promotional activities have since diminished in Japan, the situation was similar to that of Nepal as recently as 10 years ago. These activities may therefore have contributed to the high adoption rate of cephalosporins in Japan’s medical facilities. In our previous analysis of AMU in 31 Japanese hospitals, we found that all the study hospitals had adopted the use of oral third-generation cephalosporins [22]. Furthermore, most of these cephalosporins were Japanese-origin drugs that are not commonly used outside of Japan [17].

References (References 21 and 22 have been added.)

17. Tsutsui A, Yahara K, Shibayama K. Trends and patterns of national antimicrobial consumption in Japan from 2004 to 2016. J Infect Chemother. 2018;24: 414-21. doi:10.1016/j.jiac.2018.01.003

20. Amari Y, Uehara Y, Watanabe Y, Inui A, Sugihara E, Yokokawa H, et al. Status of antimicrobial use among dentists in Japan. *J Gen Hosp Med.* 2014;6: 8-15.

21. Koju P, Rousseau SP, Van der Putten M, Shrestha A, Shrestha R. Advertisement of antibiotics for upper respiratory infections and equity in access to treatment: a cross-sectional study in Nepal. *J Pharm Policy Pract.* 2020;13(4). doi:10.1186/s40545-020-0202-1

22. Kusama Y, Muraki Y, Mochizuki T, Kurai H, Gu Y, Ohmagari N. Relationship between drug formulary and frequently used cephalosporins, macrolides and quinolones in Japanese hospitals. *J Infect Chemother.* 2020;26(2):211-215. doi:10.1016/j.jiac.2019.08.013

31. Koizumi R, Kusama Y, Ishikane M, Tanaka C, Ono A, Gu Y, et al. Cross-sectional study to clarify the status of antimicrobial prescribing at outpatient care among dentists. *Kansenshogaku Zasshi.* (in press)

*2. In that regard the authors should consider mention of a couple of relatively recent studies of dental antibiotic prescribing practice from Australia (Teoh L, Stewart K, Marino R J, McCullough M J. Part 1. Current prescribing trends of antibiotics by dentists in Australia from 2013 to 2016. Aust Dent J 2018) and the USA (Durkin M J, Hsueh K, Sallah Y H et al. An evaluation of dental antibiotic prescribing practices in the United States. J Am Dent Assoc 2017; 148: 878–886.)*

Thank you for your suggestion. We have added the suggested studies of dental antibiotic prescribing practice to the Discussion section.

(Before: Discussion, Page 14, Line 201)

The prevalent use of cephalosporins by Japanese dentists is in contrast to the utilization patterns in Germany and the US [5, 21]. A national survey of German dentists showed amoxicillin to be the most commonly prescribed antimicrobial (45.8% of total AMU in 2015) [5], and a study of dental-related emergency department visits in the US revealed that penicillins accounted for over 60% of total AMU [21].

(After: Discussion)

The prevalent use of cephalosporins by Japanese dentists is in contrast to the utilization patterns in other countries [4, 5, 23-26]. For example, a national survey of German dentists showed amoxicillin to be the most commonly prescribed antimicrobial (45.8% of total AMU in 2015) [5]. A Canadian study using a population-based prescribing database reported on the dominant use of amoxicillin (1.26 DID out of 1.59 DID; 79.2% of total AMU in 2013) [4]. Similar findings have also been reported in Kuwait [23] and Scotland [24]. In comparisons of antimicrobial prescription rates, amoxicillin was also found to be the dominant antimicrobial in the US (57% in 2015) [25] and Australia (64.3% in 2016) [26].

References (References 23–26 have been added.)

4. Marra F, George D, Chong M, Sutherland S, Patrick DM. Antibiotic prescribing by dentists has increased Why? *J Am Dent Assoc.* 2016;147: 320-7. doi:10.1016/j.adaj.2015.12.014

5. Halling F, Neff A, Heymann P, Ziebart T. Trends in antibiotic prescribing by dental practitioners in Germany. *J Cranio-Maxillofacial Surg*. 2017;45: 1854-9. doi:10.1016/j.jcms.2017.08.010

23. Salako NO, Rotimi VO, Adib SM, Al-Mutawa S. Pattern of antibiotic prescription in the management of oral diseases among dentists in Kuwait. *J Dent*. 2004;32(7):503-509. doi:10.1016/j.jdent.2004.04.001

24. Roy KM, Bagg J. Antibiotic prescribing by general dental practitioners in the Greater Glasgow Health Board, Scotland. *Br Dent J*. 2000;188(12):674-676. doi:10.1038/sj.bdj.4800574

25. Durkin MJ, Hsueh K, Sallah YH, et al. An evaluation of dental antibiotic prescribing practices in the United States HHS Public Access. *J Am Dent Assoc*. 2017;148(12):878-886. doi:10.1016/j.adaj.2017.07.019

26. Teoh L, Stewart K, Marino RJ, McCullough MJ. Current prescribing trends of antibiotics by dentists in Australia from 2013 to 2016. Part 1. *Aust Dent J*. 2018;63(3):329-337. doi:10.1111/adj.12622

*3. One of the limitations of this type of study is that it cannot distinguish the reason/purpose for which the antibiotic was prescribed - and this should be mentioned in the limitations section.*

Thank you for the advice. We think that the major purpose of antimicrobials in our study setting is the prevention of infective endocarditis (IE). If oral medications are available, the Japanese Circulation Society Guidelines (2017) [33] recommend amoxicillin for IE prophylaxis; for patients with penicillin allergy, other antimicrobials such as clindamycin, azithromycin, or clarithromycin should be used. However, due to the nature of our database, we were unable to ascertain the detailed reason/purpose for which each antimicrobial was prescribed. To further investigate the reasons for prescribing antimicrobials by dentists, we are currently conducting a large-scale questionnaire-based epidemiological study. As advised, we have addressed this limitation in the revised manuscript.

(Before: Limitation, Page 16, Line 248)

This study has several limitations. First, the retrospective use of claims data means that we were unable to collect clinically relevant information regarding the prescriptions, such as patient and dentist characteristics, purpose of each prescription (therapeutic or prophylactic), and targeted diseases and procedures. Second, the data were collected only from the NDB, which did not include uninsured patients such as tourists.

(After: Limitation)

This study has several limitations. First, the retrospective use of claims data means that we were unable to collect clinically relevant information regarding the prescriptions, such as patient and dentist characteristics, purpose of each prescription (therapeutic or prophylactic), and targeted diseases and procedures. Japan’s Guidelines for the Clinical Management of Infectious Diseases [29] and the Japanese Circulation Society’s 2017 Guidelines on the Prevention and Treatment of Infective Endocarditis [33] recommend the use of antimicrobials for infective endocarditis prophylaxis, which may represent one of the main reasons for antimicrobial prescriptions by dentists. Nevertheless, we were unable to ascertain the specific reason for which each antimicrobial was prescribed. We are currently conducting a questionnaire-based epidemiological study to further investigate the purpose of antimicrobial prescriptions by dentists. Second, the data were collected only from the NDB, which did not include uninsured patients such as tourists.

References (Reference 33 has been added.)

29. Kaneko A, Aoki T, Ikeda F, Kawabe R, Satoh T, Tsumura N. The 2016 JAID/JSC guidelines for clinical management of infectious disease−Odontogenic infections. *J Infect Chemother.* 2018;24: 320-4. doi:10.1016/j.jiac.2017.09.014

33. Nakatani S. Guideline on Prevention and Treatment of Infective Endocarditis. *Circ J.* 2019;83:1767-1809. doi:10.1253/circj.CJ-19-0549

*4. The current categorisation of antibiotics into such broad categories seems rather a blunt instrument. Are the investigators not able to provide a breakdown of the types of penicillins - at least Amoxicillin v other penicillins to allow better comparison with the findings in other countries and a better understanding of compliance with Japanese AB and AP prescribing guidelines? Similarly, for comparison with other international studies data on the prescribing of clindamycin would be very helpful - if available.*

Thank you for your suggestion. We agree that the current categorization is rather broad. If we collected more detailed data on the classification of antimicrobials, it may be possible to compare the use of amoxicillin and clindamycin (in addition to cephalosporins) with that of other countries. This may even help to identify the factors other than the promotional activities of pharmaceutical companies that explain why cephalosporins are the most commonly prescribed antimicrobial among Japanese dentists. However, our study was based on ATC Level 4 codes, which made it difficult to identify ampicillin and clindamycin use. The identification of ampicillin and clindamycin requires ATC Level 5 codes, in which amoxicillin is coded as J01CA04 and clindamycin is coded as J01FF01. The “penicillins” in our study comprised a combination of three sub-categories (J01CA, J01CE, and J01CR) at ATC Level 4; here, amoxicillin belongs to J01CA. The DID values of J01CA—including amoxicillin—were 0.10 in 2015, 0.10 in 2016, and 0.12 in 2017. These values were almost equivalent to those of “penicillins” in our study. Similarly, clindamycin belongs to J01FF at ATC Level 4. The DID values of J01FF—including clindamycin—were 0.0017 in 2015, 0.0019 in 2016, and 0.0022 in 2017. We have added these results to the Results section, and hope this helps provide insight for readers to make any comparisons if needed.

(Before: Result, Page 10, Line 155)

In contrast, the AMU trends for macrolides and fluoroquinolones were stable over time. Table 2 presents the DID values of total AMU among dentists stratified by administration route.

(After: Result)

In contrast, the AMU trends for macrolides and fluoroquinolones were stable over time. The penicillins in Table 1 included antimicrobials with ATC codes J01CA, J01CE, and J01CR. The DID values of J01CA antimicrobials (which include amoxicillin) were 0.10 in 2015, 0.10 in 2016, and 0.12 in 2017. The DID values of J01FF antimicrobials (which include clindamycin) were 0.0017 in 2015, 0.0019 in 2016, and 0.0022 in 2017. Table 2 presents the DID values of total AMU among dentists stratified by administration route.

**Reviewer #2’s Comments:**

*1. You might add a sentence about veterinarian and the fact that they are doctor and pharmacologist in personal union, at least in some countries. The stewardship should reach them also and quickly.*

Thank you for your thoughtful and constructive feedback regarding our manuscript.

We agree with your suggestion. While physicians and dentists are the main ASP targets in human medicine, veterinarians are also a vital target for AMR in animal medicine under the WHO’s One Health approach. As advised, we have added the suggested information to the Discussion section with an additional reference.

(Before: Discussion, Page 17, Line 254)

To the best of our knowledge, this is the first nationwide study of the epidemiology of AMU among Japanese dentists.

(After: Discussion)

To the best of our knowledge, this is the first nationwide study of the epidemiology of AMU among Japanese dentists. In addition to dentists and physicians, AMU should also be examined in veterinarians as they act as both physicians and pharmacists in Japan and other countries. As the World Health Organization’s One Health approach recommends a holistic and multisectoral approach to AMR measures, there is an urgent need to implement effective ASPs for both human and veterinary medicine [34].

References (Reference 34 has been added.)

34. World Health Organization. Global Action Plan on Antimicrobial Resistance. Vol 10.; 2015. Accessed November 25, 2020. https://www.who.int/antimicrobial-resistance/global-action-plan/en/

*2. The second comment is related to this impressively high prescription rate of cephalosporins in Japan (above 60%). In other countries it is listed “among others” with only a few prescriptions at dental practices. To avoid such false prescriptions in the future, it is relevant to track the origin of problem. For instance, in our country clindamycin was (and is) a blockbuster in dentistry. The simple reason is the intense promotion by the corresponding manufacturer. That means, antibiotics of most attention (not of most efficacy) are prescribed. What could be the reason for such an over-prescription in Japan? It is known that there is cross-allergy between penicillins and cephalosporins (about 20%). My hypothesis is that the origin is related to allergy rates, namely that cephalosporins are generally regarded as safer? You might proof this hypothesis.*

Thank you very much for your comments and hypothesis. As you pointed out, cephalosporins may be prescribed if there are concerns for penicillin allergy. However, cephalosporins are not recommended as the primary alternative drug for such cases in Japan’s guidelines. We agree that a possible reason for the preference for cephalosporins in Japan’s medical facilities may be due to the active promotion of these antimicrobials by pharmaceutical companies. A recent study reported that the top-selling antimicrobials in Nepal were those with a higher number of promotional activities [21]. Although such activities have since diminished in Japan, the situation was similar to that of Nepal until only 10 years ago. These promotions may have contributed to the high adoption rate of cephalosporins in medical facilities in Japan when compared to those in other countries. In fact, we have previously shown in an analysis of 31 Japanese hospitals that all the study hospitals had adopted the use of oral third-generation cephalosporins [22]. Additionally, most of these cephalosporins were Japanese-origin drugs that are not commonly used outside of Japan. Nevertheless, we recognize that this is a hypothesis, and needs further empirical investigation. Therefore, based on the results of our previous questionnaire-based study on AMU among dentists in Japan [31], we are currently conducting a nationwide questionnaire-based epidemiological study to determine the factors (including the perceived safety of cephalosporins with respect to allergy rates) that influence prescriptions of third-generation cephalosporins among 1700 dentists throughout the country. We have added this information to the Discussion section.

(Before: Discussion, Page 14, Line 201)

These results corroborate those of a previous cross-sectional questionnaire-based study on dentists at community clinics in Japan [20].

(After: Discussion)

These results corroborate those of a previous cross-sectional questionnaire-based study on dentists at community clinics in Japan [20]. The frequent use of cephalosporins in Japan may be due in part to the active promotion of these antimicrobials by pharmaceutical companies. A study conducted in Nepal showed that the top-selling antimicrobials were those that were subjected to intensive promotional activities [21]. Although such promotional activities have since diminished in Japan, the situation was similar to that of Nepal as recently as 10 years ago. These activities may therefore have contributed to the high adoption rate of cephalosporins in Japan’s medical facilities. In our previous analysis of AMU in 31 Japanese hospitals, we found that all the study hospitals had adopted the use of oral third-generation cephalosporins [22]. Furthermore, most of these cephalosporins were Japanese-origin drugs that are not commonly used outside of Japan [17].

References (References 21 and 22 have been added.)

17. Tsutsui A, Yahara K, Shibayama K. Trends and patterns of national antimicrobial consumption in Japan from 2004 to 2016. *J Infect Chemother.* 2018;24(6):414-421. doi:10.1016/j.jiac.2018.01.003

20. Amari Y, Uehara Y, Watanabe Y, Inui A, Sugihara E, Yokokawa H, et al. Status of antimicrobial use among dentists in Japan. *J Gen Hosp Med.* 2014;6: 8-15.

21. Koju P, Rousseau SP, Van der Putten M, Shrestha A, Shrestha R. Advertisement of antibiotics for upper respiratory infections and equity in access to treatment: a cross-sectional study in Nepal. *J Pharm Policy Pract.* 2020;13(4). doi:10.1186/s40545-020-0202-1

22. Kusama Y, Muraki Y, Mochizuki T, Kurai H, Gu Y, Ohmagari N. Relationship between drug formulary and frequently used cephalosporins, macrolides and quinolones in Japanese hospitals. *J Infect Chemother.* 2020;26(2):211-215. doi:10.1016/j.jiac.2019.08.013

31. Koizumi R, Kusama Y, Ishikane M, Tanaka C, Ono A, Gu Y, et al. Cross-sectional study to clarify the status of antimicrobial prescribing at outpatient care among dentists. *Kansenshogaku Zasshi.* (in press)
